# Association of hyperactivated transposon expression with exacerbated immune activation in systemic lupus erythematosus

**DOI:** 10.1186/s13100-024-00335-8

**Published:** 2024-10-19

**Authors:** Frank Qingyun Wang, Xiao Dang, Huidong Su, Yao Lei, Chun Hing She, Caicai Zhang, Xinxin Chen, Xingtian Yang, Jing Yang, Hong Feng, Wanling Yang

**Affiliations:** Department of Paediatrics and Adolescent Medicine, The University of Hong Kong, Hong Kong, China

**Keywords:** Systemic lupus erythematosus, Interferon, Nucleic acid sensor, Transposon expression

## Abstract

**Background:**

Systemic Lupus Erythematosus (SLE) is a complex autoimmune disorder, and transposable elements (TEs) have been hypothesized to play a significant role in its development. However, limited research has explored this connection. Our study aimed to examine the relationship between TE expression and SLE pathogenesis.

**Methods:**

We analyzed whole blood RNA-seq datasets from 198 SLE patients and 84 healthy controls. The REdiscoverTE pipeline was employed to quantify TE and other gene expressions, identifying differentially expressed TEs. A TE score was calculated to measure overall TE expression for each sample. Gene ontology and gene set enrichment analyses were conducted to explore the functional implications of TE upregulation. Independent datasets were utilized to replicate the results and investigate cell type-specific TE expression.

**Results:**

Our analysis identified two distinct patient groups: one with high TE expression and another with TE expression comparable to controls. Patients with high TE expression exhibited upregulation of pathways involving nucleic acid sensors, and TE expression was strongly correlated with interferon (IFN) signatures. Furthermore, these patients displayed deregulated cell composition, including increased neutrophils and decreased regulatory T cells. Neutrophils were suggested as the primary source of TE expression, contributing to IFN production.

**Conclusions:**

Our findings suggest that TE expression may serve as a crucial mediator in maintaining the activation of interferon pathways, acting as an endogenous source of nucleic acid stimulators in SLE patients.

**Supplementary Information:**

The online version contains supplementary material available at 10.1186/s13100-024-00335-8.

## Background

Systemic Lupus Erythematosus (SLE) is a complex autoimmune condition characterized by widespread immune dysregulation. Currently, there is no definitive cure for SLE, and patients often experience cycles of relapse and remission [1]. This chronic and unpredictable condition presents significant challenges in terms of diagnosis and management. The continuous activation of the immune system in SLE is believed to be influenced by persistent intrinsic stimuli, also known as autoantigens, which consistently existed and can't be eliminated from the patients [2–4]. Both the innate and adaptive immune systems are involved in this condition, including the production of autoantibodies and abnormal induction of type I interferon (IFN) [5, 6]. In this study, we focused on investigating the potential role of transposable elements (TEs), which are the most abundant self-nucleic acids, in the pathogenesis of SLE.

Transposable elements (TEs) comprise nearly half of the human genome and can be classified into two main classes: Class I, also referred to as endogenous retrotransposons, replicate using an RNA intermediate and require reverse transcriptase for integration into the genome. They can be further categorized into two distinct groups: human endogenous retroviruses (HERVs) with long terminal repeats (LTRs), and those without them, known as long interspersed elements (LINEs) and short interspersed elements (SINEs). Class II TEs, or DNA transposons, replicate through a “cut and paste” manner. Throughout evolutionary history, most TEs have accumulated mutations, causing them to lose their ability to replicate.

To date, only L1HS from the LINE subfamily can translate functional reverse transcriptase and independently replicate. Several studies have revealed that SLE patients produce auto-antibodies against the ORF1p encoded by L1HS [7–10]. Their presence has been linked to immune cell activation, but their relationship with disease activity remains elusive. A small portion of ERVs have relatively intact genome structure with some or full open reading frames (ORFs). Researchers found that ERV-K102 encoded an intact ORF that could produce virus envelope protein, involved in the formation of immune complexes and contribute to neutrophil activation [11]. In addition to the potential impact on the immune system from the protein generated by the transposable elements, long terminal repeats found within HERVs can also serve as regulatory elements and exert control over the expression of genes in close proximity [12, 13].

Importantly, although most TEs have lost the ability to translate proteins, nucleic acids resulting from the transcription of transposable elements could be recognized by innate immune sensors termed pattern recognition receptors (PRRs). Since transposon-derived products resemble viral-like DNA and RNA, they are likely to activate these PRRs through a mechanism known as "viral mimicry" [14, 15]. Toll-like receptors (TLRs), which are located on the cell membrane or within endosomes, are capable of detecting double-stranded RNA (dsRNA), single-stranded RNA (ssRNA), single-stranded DNA (ssDNA), as well as RNA/DNA hybrids. Furthermore, the cytoplasmic and nuclear receptors MDA5 and RIG-I can recognize both long and short dsRNA molecules. Downstream of these PRRs, the NF-kB pathways and Interferon regulatory factors (IRFs) will be activated, promoting the production of pro-inflammatory cytokines, chemokines, and Type I IFNs.

Under normal conditions, transposable elements (TEs) activities in the human genome are largely suppressed through epigenetic mechanisms such as DNA methylation and histone modifications [16, 17]. In Aicardi-Goutières syndrome, a rare genetic disorder sharing features with SLE, defects in regulating transposon activities have been connected with the IFN pathways [18, 19]. Recent studies have also suggested that aberrant expression of TEs may occur in SLE [20–22]. The dysregulation of methylation has been indicated as the possible mechanism by which transposons are upregulated in SLE patients [23, 24]. Additionally, studies have shown an interaction between autoantibodies and RNA-binding protein Ro-60 with SINEs [25]. Nevertheless, these studies have been limited by relatively small sample sizes and have not generated a clear pattern of TE expression in SLE. In the present study, we integrated multiple whole blood RNA-seq datasets from 198 SLE patients and 84 healthy controls (HCs) to investigate TE expression in SLE. We identified the most deregulated TE subfamilies and established an association between innate immune dysregulation and TE expression. Furthermore, we quantified TE expression in different cell types and observed a similar pattern in polymorphonuclear neutrophils.

## Methods

### Selection of dataset

We obtained raw sequencing data (.fastq) from the Gene Expression Omnibus (GEO). The whole blood RNA-seq datasets used in this study were retrieved with the following accessions: PRJNA294187 (SLE: 99, HC: 18) [25], PRJNA318253 (SLE: 12, HC: 4) [26], PRJNA439269 (SLE: 31, HC: 28) [27], PRJNA717024 (SLE: 24, HC: 23) [21], and PRJNA921887 (SLE: 32, HC: 11) [28]. To validate and replicate the relationship between IFN score and TE expression, we utilized an independent whole blood RNA-seq dataset, PRJNA476781 (SLE: 175) [29]. To investigate cell type-specific TE expression, we utilized a cell type-specific RNA-seq dataset with the accession PRJNA627214 [30].

### Analysis of gene and TE expression

We employed the REdiscoverTE pipeline to measure both gene expression and subfamily level TE expression, as previously described [31]. To minimize the potential confounding between TE-derived reads and gene-derived reads, we selected the intergenic TE expression output from REdiscoverTE for downstream analysis. Our analysis focused solely on the Long-Interspersed Element (LINE), Short-Interspersed Element (SINE), DNA Transposon, Long Terminal Repeat (LTR), and Retroposon TE subfamilies. Raw counts were normalized using the edgeR algorithm, which accounted for library size, and log2CPM was calculated using a prior count set as 1 [32]. For downstream analysis, we utilized the limma::removeBatchEffect function to remove the batch effect of the normalized log2CPM counts. We used the disease conditions of the samples as the design matrix to preserve the actual features across the conditions, and the dataset origin of the samples as the batch matrix [33]. Through principle component analysis (PCA), we identified and removed one outlier (Supplementary Fig. 1B-D). We utilized the edgeR algorithm to identify differentially expressed TE and genes between the conditions [32], with experimental batch and conditions added as covariates in the model matrix to optimize theanalysis. Our screening criteria for differentially expressed TE and genes were FDR < 0.05 and log2FC > 0.5.

### Classification of SLE patients

We applied Ward’s method using R pheatmap package to hierarchically cluster the patient samples into two groups based on upregulated TEs that was identified through the analysis of differential expression. We then calculated a score for each subject to measure the overall expression level of TE. Specifically, we calculated the z-score for each upregulated TE. These z-scores were then summed in each individual to obtain the TE score.

### Functional analysis

We conducted Gene Ontology (GO) enrichment analysis on the up-regulated and down-regulated genes separately, comparing TE high, TE low, and healthy controls. This analysis was performed using the R package clusterProfiler [34]. Additionally, we used Gene Set Enrichment Analysis (GSEA) to analyze the gene list ranked by the log2FC from the edgeR results between the TE high and TE low groups. Moreover, Gene Set Variation Analysis (GSVA) was performed on the normalized batch-corrected gene expression data. This analysis assigns gene set enrichment scores for the target pathways for each sample. These analysis employed Kyoto Encyclopedia of Genes and Genomes (KEGG) and Reactome terms, obtained from MSigDB [35].

### Cell deconvolution analysis

We used the CIBERSORTx algorithm to deduce the relative abundance of immune cells from RNA-seq data obtained from whole blood. The Leukocyte signature matrix (LM22), which includes 547 genes, was employed for this purpose [36]. The analysis was conducted on batch-corrected normalized gene expression data. To compare the cell proportions between TE high, TE low, and healthy controls, we used the Wilcoxon signed-rank test, which was implemented in the R ggpubr package. The test was based on the relative proportion of each cell type.

### Statistical analysis

All statistical analyses were performed using the R software. The IFN gene sets used in this study were selected from a previous research, which categorized the IFN genes into three distinct groups (M1.2, M3.4, M5.12) with different characteristics based on the coexpression analysis [37]. The IFN score was calculated using the same method as described earlier for the TE score. To establish the correlation between the TE expression with GSVA score/IFN expression, a linear regression model implemented in R as lm() was employed.

## Results

### TE expression are hyperactivated in SLE

**Fig. 1:**
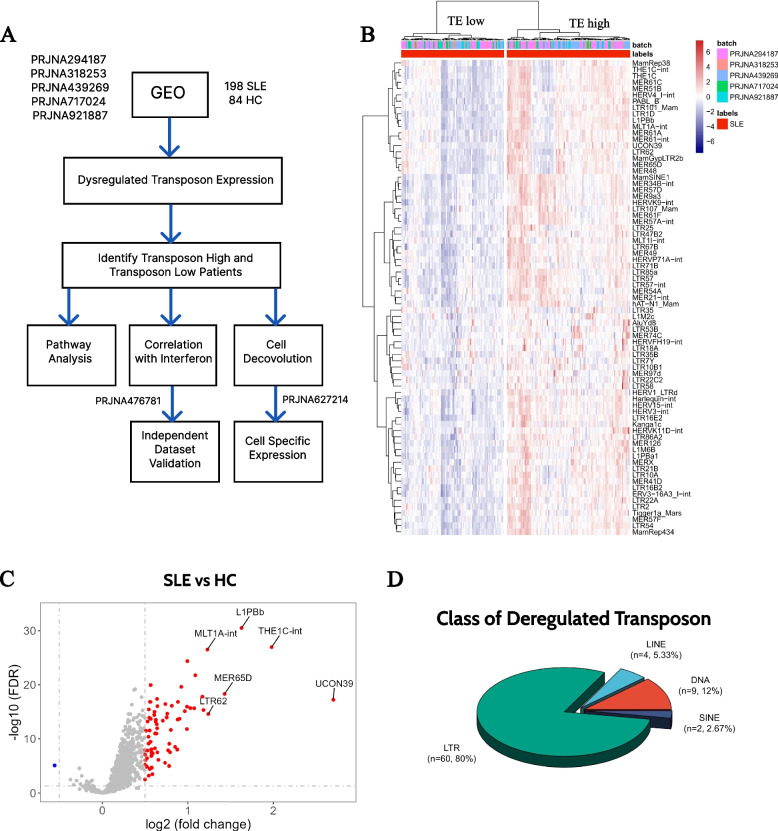
Landscape of TE expression in SLE. **A** The flow chart of the analysis describing the study design and the datasets used. **B** Volcano plot of differentially expressed TEs between SLE patients and healthy controls (Up: 75, Down: 1). FDR < 0.05 and log2 Fold Change > 0.5 were used as cut off to screen differentially expressed TEs. **C** Heatmap of the 75 upregulated TEs in SLE. The dendrogram showcase the SLE patients can be clustered into two groups. **D** Pie chart shows the class of the 75 upregulated TEs (LTR: 80%, LINE: 5.33%, DNA: 12%, SINE: 2.67%)

We performed an integrative analysis of five publicly available whole blood RNA-seq datasets obtained from the GEO database, which included 198 SLE patients and 84 healthy controls (Fig. 1A). Our analysis focused on quantifying the expression of over 1,000 transposable TEs located in intergenic regions at the subfamily level. We aimed to explore the expression landscape of transposons and identify differentially expressed transposons between SLE patients and healthy controls. Our results revealed a significant increase in TE expression in SLE patients, with 75 transposons showing upregulation and only one transposon showing downregulation (Fig. 1C). Among the 75 upregulated transposons, LTR retrotransposons accounted for the majority (80%), followed by DNA transposons (12%), LINEs (5.33%), and SINEs (2.67%) (Fig. 1D). Additional information about these TEs are annotated based on Dfam database and a public dataset that describe TE’s potential to form dsRNA [38, 39]. It is suggested that out of the 75 TEs examined, 41 of them have at least one locus in the genome with the potential to form dsRNA. Additionally, 10 of the 75 TEs have a curated coding sequence in Dfam. (Supplementary Table 1).

### TE expression distinguishes SLE patients with different molecular features

**Fig. 2:**
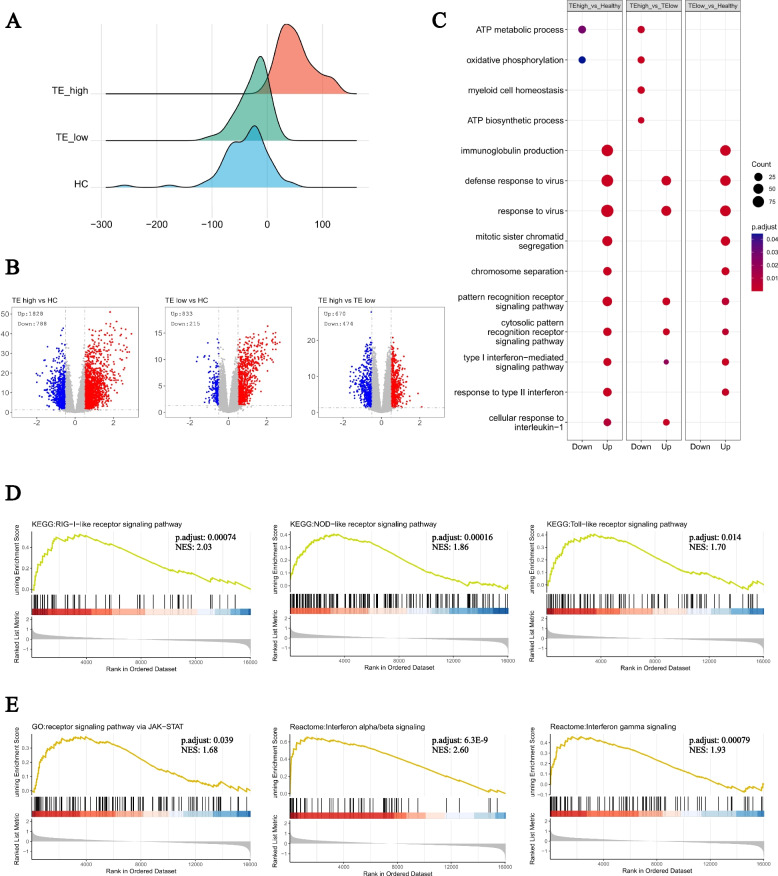
TE Expression Distinguishes SLE Patients with Different Molecular Features. **A** Ridge plot shows the TE score in three groups (HC vs TE low vs TE high). **B** Volcano plot of the differentially expressed genes among three groups (Left: TE high vs HC, Middle: TE low vs HC, Right: TE high vs TE low). **C** Dotplot shows the Gene Ontology (GO) enrichment results of the up- and down-regulated genes among the three groups (Left: TE high vs HC, Middle: TE high vs TE low, Right: TE low vs HC). **D**, **E** GSEA enrichment plot shows the enrichment score of the IFN related pathways in the TE high patients comparing to TE low patients

Considering the observed global up-regulated trend of transposable element TE expression, we conducted further investigations to determine whether TE expression exhibits a heterogeneous pattern within the patient population. Using a hierarchical clustering method, we classified 108 SLE patients as TE-high and 90 patients as TE-low (Figs. 1B and 2A). Interestingly, a larger number of genes were dysregulated in TE-high patients when compared to healthy controls (Upregulated: 1828, Downregulated: 788) than in TE-low patients (Upregulated: 833, Downregulated: 215) (Fig. 2B).

The GO analysis indicated that both TE-high and TE-low groups exhibited upregulated immune pathways such as "immunoglobulin production", "response to virus", "type I interferon-mediated signaling pathway", and "cytosolic pattern recognition receptor signaling pathway". Furthermore, these pathways were more significantly upregulated in TE-high patients than TE-low patients. Conversely, pathways, including "myeloid cell homeostasis", "oxidative phosphorylation" and "ATP metabolic process", were downregulated in TE-high patients comparing to the TE low group (Fig. 2C).

We furtherly performed GSEA to validate that innate immune responses, particularly those related to interferon signaling, were more activated in TE-high patients compared to TE-low patients. Pathways such as "KEGG: NOD-like receptor signaling pathway", "KEGG: Toll-like receptor signaling pathway", and "KEGG: RIG-I-like receptor signaling pathway" were upregulated in TE-high patients, which are involved in sensing cytosolic RNA and DNA that could be products of transposon expression (Fig. 2D). Downstream pathways, including "GO: receptor signaling pathway via JAK-STAT", "Reactome: Interferon alpha/beta signaling", and "Reactome: Interferon gamma signaling", were also upregulated in TE-high patients (Fig. 2E).

### Association between TE score and interferon expression

**Fig. 3:**
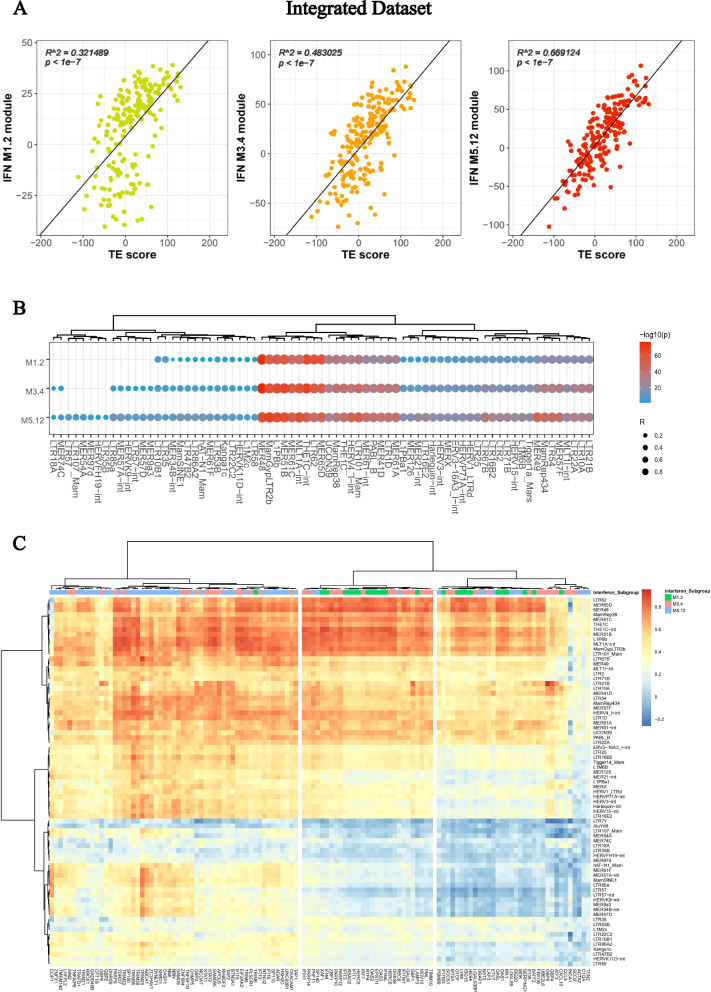
TE expression is correlated with IFN level in SLE. **A** Correlation between the three IFN expression modules and TE score in the integrated dataset (Left: M1.2, Middle: M3.4, Right: M5.12). **B** Bubble plot shows correlation between the expression of each TE subfamily with the three IFN modules of in the integrated dataset (Left: M1.2, Middle: M3.4, Right: M5.12). **C** Heatmap showcasing the correlation between the expression of each TE subfamily and the individual genes within the three IFN modules. Color represents R value derived from Pearson correlation

In a previous study, it was reported that upregulated interferon expression could be classified into three distinct sets, each with a unique activation threshold (M1.2 < M3.4 < M5.12) [37]. We investigated the correlation between the TE score and IFN score in each module and found that all three IFN modules exhibited a significant correlation with TE expression (p < 1e-7). However, we noted that the M1.2 module had the weakest correlation (R^2: 0.32), the M3.4 module had a moderate correlation (R^2: 0.48), and the M5.12 module had the strongest correlation (R^2 = 0.67) (Fig. 3A). We subsequently dissected this correlation on the TE subfamily level, it is suggested that most of the TE subfamilies contribute to the correlation with IFN expression. We discovered a broader range of TEs whose expression is significantly associated with IFN genes in the M5.12 module compared to those in the M3.4 and M1.2 modules. Additionally, different TEs have varying levels of correlation with the IFN signature, with a subset of TEs, including MER48, TH1C, and HERV4, exhibiting the strongest correlation to the IFN signature (Fig. 3B, C). In addition to correlating the TE with general IFN expression, our GSVA analysis demonstrated that subfamily TE expression is also significantly correlated with pathways such as "TRAF6-mediated IRF7 activation", "Interleukin 6 signaling", and "Met activates PI3K-AKT signaling" (Supplementary Fig. 2).

To validate these findings, we analyzed the expression of transposable elements and interferons in an independent dataset of 175 patients before an anti-IL-6 drug clinical trial [29]. Similar to our initial observations, we found that TE expression had a stronger correlation with M5.12 (R^2: 0.50) compared to M3.4 (R^2: 0.46) and M1.2 (R^2: 0.38) (Supplementary Fig. 3A). On the subfamily level, these TEs remains significantly correlated with the genes in the three modules, but also exhibited heterogeneity (Supplementary Fig. 3B, C). The relationship between the expression of TE subfamilies and IFN is concordant among the two datasets (Supplementary Fig. 3D). This correlation pattern was consistent with the different activation thresholds of the three modules, suggesting an association between transposable element expression and interferon activation.

### Immune cell composition is correlated with TE expression

**Fig. 4:**
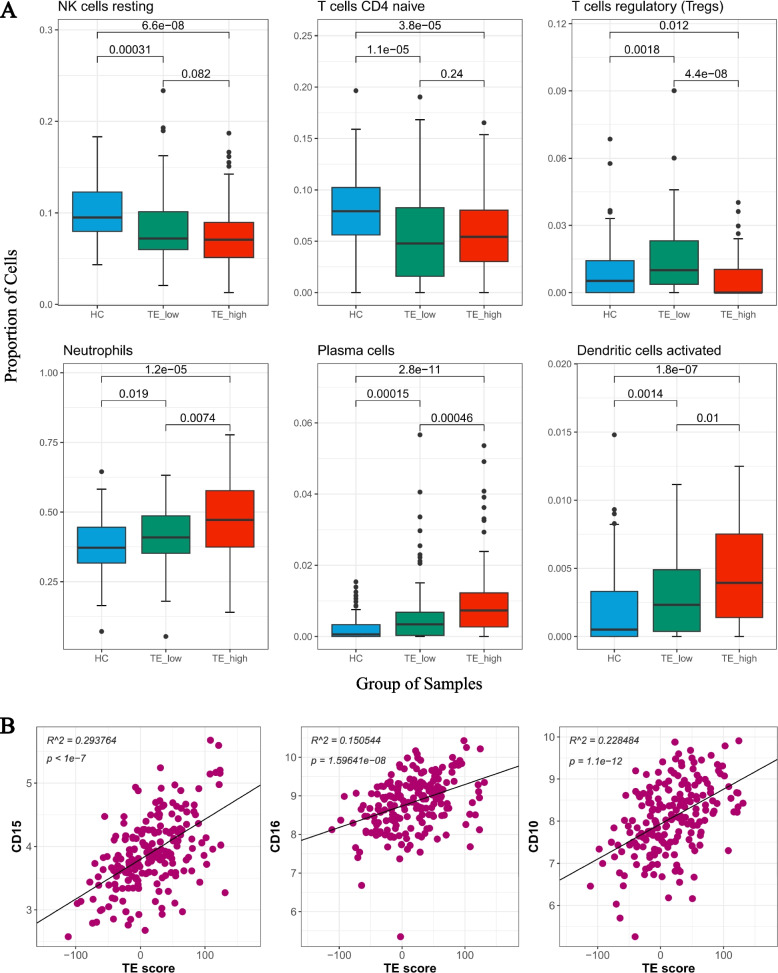
TE expression is associated with the cell composition in SLE patients. **A** Boxplots show the proportions of various cell types among HC, TE low and TE high patients (Up panel: NK cells resting, T cells CD4 naïve, T cells regulatory. Down panel: Neutrophils, Plasma cells, Dendritic cells activated). *P*-value result from the Wilcoxon signed-rank test. **B** Correlation between certain genes and TE score

After establishing the relationship between TE expression and innate immune response in SLE, we aimed to investigate the relationship between TE expression and immune cell composition. We utilized CibersortX to dissect the cell composition in TE high patients, TE low patients, and healthy controls. Our analysis revealed that the proportions of resting NK cells and naive CD4 T cells were similarly downregulated in both TE high and TE low groups, reflecting the general lymphopenia state observed in SLE patients. Notably, the proportion of regulatory T cells was lower in TE high patients than in TE low patients, which is consistent with the more deregulated immune activation state observed. Furthermore, the proportions of neutrophils, plasma cells, and activated dendritic cells were significantly higher in TE high patients compared to both TE low patients and healthy controls (Fig. 4A). Our analysis also revealed a positive correlation between the TE score and the expression of CD15, CD16, and CD10, which are common markers found on neutrophils (Fig. 4B). These findings support the notion that TE expression may associate with the abundance of the immune cells in SLE patients, potentially involved in the disease's pathogenesis.

### Dysregulation of TE expression is prominent in neutrophils

**Fig. 5:**
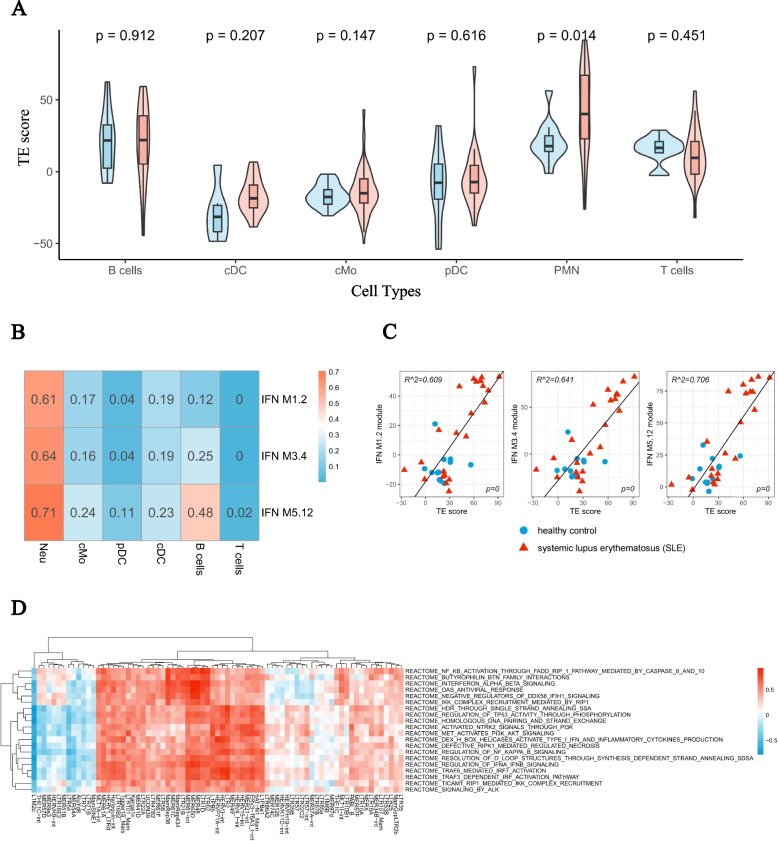
Association between TE expression and IFN is prominent in SLE PMN cells comparing to other cell types. **A** Boxplots show the TE score among different cell types in SLE and healthy controls. **B** Heatmap shows the correlation between TE score and three modules of IFN genes in various cell types. Number denotes the value of R^2 in linear regression. **C** Correlation between the TE score and three modules of IFN in PMNs. **D** Correlation plots depict the expression of each TE subfamily in relation to the GSVA Reactome pathway score in neutrophils. The pathways exhibiting the highest average correlation with the 75 TEs are illustrated, with color indicating the R value derived from Pearson correlation

We further investigated cell type-specific TE expression using an independent dataset that utilized bulk RNA-seq on flow cytometry-separated B cells, T cells, conventional dendritic cells (cDC), classical monocytes (cMo), plasmacytoid dendritic cells (pDC), and polymorphonuclear neutrophils (PMN) [30]. Utilizing the 75 previously identified upregulated transposons, we calculated the TE score for each cell type-specific sample and found that PMNs exhibited the highest score among all cell types. More importantly, SLE PMNs exhibited significantly higher TE scores than healthy PMNs (Fig. 5A). The TE score exhibited the strongest correlation with the three IFN modules in PMNs. Moderate correlations were observed in B cells, cMo, and cDC, while little or no correlation was observed in pDC and T cells. The trend of increased correlation with the three IFN modules was also observed in PMNs (IFN M1.2: 0.61, IFN M3.4: 0.64, IFN M5.12: 0.71) and B cells (IFN M1.2: 0.12, IFN M3.4: 0.25, IFN M5.12: 0.48), which is consistent with previous findings from whole blood analysis (Fig. 5B, 5C). Focusing on the neutrophils, the functional analysis indicates the pathways which have strongest correlation with TEs on the subfamily level includes multiple pathways involved in the interferon activation such as “TRAF6 mediated IRF7 activation”, “OAS antiviral response”, “Negative regulators of DDX58 IFIH1 signalling”.

## Discussion

Our integrated analysis of 198 SLE patients and 84 HCs revealed a global activation of intergenic TE expression in SLE patients. While the upregulated trend of TE expression is prominent in SLE patients, we acknowledged the heterogeneity within the patient population and thus divided them into two subgroups based on the pattern of TE expression. Functional analysis between these two groups revealed that the subgroup with high TE expression exhibited an activated innate immune response, particularly related to the IFN pathways.

Upstream of the IFN pathways, we observed activation of nucleic sensors such as the NOD-like receptor, Toll-like receptor, and RIG-I-like receptor in the TE high group. Extensive research has been conducted on the relationship between these cytosolic nucleic acid sensors and SLE [40]. TLR7, for example, recognizes intracellular single-stranded RNA, and GWAS studies have identified susceptible loci around the TLR7 regions [41, 42]. Furthermore, functional studies have demonstrated that a single genetic variation in TLR7 is sufficient to induce a lupus-like phenotype [43]. MDA5, which is one of the RIG-I-like receptors encoded by IFIH1 recognizing double-stranded RNA, has also been repeatedly implicated in GWAS studies [44, 45]. The susceptibility of nucleic sensors in SLE patients makes them more prone to pathogenic activation. Among the transposons, both single-stranded RNAs and double-stranded RNAs can be formed from abnormal transcriptional activities. The repetitive nature of Alu allows the formation of intramolecular dsRNA [46]. Regarding the ERVs, bi-directional LTR leads to dsRNA formation through the transcription of sense and antisense transcripts, and two adjacent ERVs in opposite orientations could also form a hairpin structure [46, 47]. It has been suggested that induction of ERV expression results in activation of the MDA5, RIG-I, and downstream innate immune response, which has been exploited in the cancer treatment [47, 48]. The theory of molecular mimicry suggests similarities between foreign and self substrates can cross-activate autoreactive immune cells. According to a public dataset, 41 out of 75 TEs identified in our study have the potential to form dsRNA in at least one locus in the genome, indicating these virus-like TEs might be sensed by the innate immunity and contribute to the prolonged inflammation observed in the patients [39].

We conducted a correlation analysis between the TE score and three pre-defined IFN groups [37]. The M1.2 IFN group exhibited the lowest activation threshold and was observed in patients with different levels of disease severity. On the other hand, the M3.4 and M5.12 groups had higher activation thresholds and showed stronger correlation with disease activity. We found that the TE score had a stronger correlation and more TE subfamilies are associated with the M5.12 group compared to the M3.4 and M1.2 groups. Furthermore, we found that a specific group of TEs contributed more significantly to the association with IFN signature compared to other subfamilies, indicating a varying degree of relationship between TE subfamilies and the IFN signature. These finding supports the idea that TE expression plays a role in the activation of the IFN pathway. Although we have limited clinical information of the curated dataset, since M5.12 module correlated well with the disease activity and TE expression correlate best with M5.12, it could be inferred that the overall transposon activities also associate with the disease activities.

Moreover, our analysis revealed that the "myeloid cell homeostasis" pathway was downregulated in TE high patients compared to TE low patients. This pathway plays a role in regulating the total number of myeloid cells by controlling proliferation and apoptosis pathways. To further investigate this finding, we utilized CibersortX to estimate cell proportions from the bulk data. Interestingly, we observed a significantly higher proportion of neutrophils in TE high patients compared to TE low patients, suggesting that TE expression is associated with neutrophil expansion. Previous studies have reported an increased expression of L1 retrotransposons in SLE granulocytes, which is attributed to a reduction in epigenetic silencing and has been correlated with disease activity [8]. Furthermore, antibodies against HERV-K have been identified in SLE plasma, forming immune complexes that activate neutrophils [11]. Consistent with these findings, our analysis investigated cell type-specific TE expression and revealed that polymorphonuclear neutrophils exhibited the strongest TE overexpression compared to other cell types in SLE patients. In addition, the expression of TE on the subfamily level is correlated with the IFN-associated pathways in neutrophils. It has been reported that neutrophils are one of the primary sources of IFN production [49, 50]. Considering that neutrophils constitute nearly half of the blood cell population, it is suggested that TE expression could couple with neutrophil activation and involve in the IFN production in SLE patients.

The increased proportion of activated dendritic cells and plasma cells provides further evidence that patients with higher TE expression exhibit both innate and adaptive immune activation. On the other hand, the decreased proportion of regulatory T cells in TE high patients compared to TE low patients is consistent with a more deregulated immune system.

The current treatment strategy for SLE involves the use of glucocorticoids and other immunosuppressants, which have a broad immunosuppressive effect. More targeted therapies could be explored to specifically address the interaction between endogenous RNA and nucleic acid sensors [51]. One example of a targeted therapy is anifrolumab, a monoclonal antibody that targets the IFN-alpha receptor downstream of the nucleic acid sensors. Clinical studies have demonstrated that anifrolumab, when used in combination with standard treatment, effectively reduces disease activity in patients with moderate-to-severe SLE [52]. Additionally, targeting nucleic acid sensors using antagonists has shown promise in the treatment of multiple autoimmune diseases, including gout, rheumatoid arthritis, and SLE, and is an area undergoing active research [53].

Our study indicates an association between the TE expression and IFN related pathways, but a causal relationship can’t be established without experimental validation. In SLE patients, significant epigenetic alterations occur within their genome, leading to the overexpression of numerous genes [23, 54]. This may include TEs, which constitute a major component of the human genome. A subset of these TEs might be able to form virus-like products, which could in-turn furtherly exacerbate IFN signalling by virial mimicry. Though TEs may not be the initial triggers of the IFN response, they could potentially play a role in maintaining and amplifying this response, thereby prolonging the disease. To better understand the relationship between TEs and exacerbated immune activation, further research is required, such as employing third-generation sequencing and conducting experiments to explore the interplay between locus-level TE expression and activated immune response in SLE.

## Conclusion

In our study, we conducted an integrative analysis of TE expression using RNA-seq data from whole blood samples consisting of 198 SLE patients and 84 healthy controls. Our analysis revealed a global upregulation of TE expression in SLE patients. Based on the levels of TE expression, we categorized the SLE patients into two groups: TE high and TE low. Notably, the TE high group showed increased activation of nucleic acid sensor pathways, elevated IFN production, and increased proportions of neutrophils together with decreased proportions of regulatory T cells. More specifically, our analysis identified neutrophils as the primary contributors to TE expression in SLE patients. These findings highlight the association between TE expression and innate immune pathways, providing insights into the role of TE expression as a potential mediator of the broad immune dysregulation observed in SLE.

## Supplementary Information

Supplementary Material 1: Supplementary Fig. 1. PCA plots of all the samples from the five datasets. (A) PCA plot prior to batch correction. (B-E) Batch effect across different datasets are minimized after limma::removeBatchEffect according the PCA plots. We identified and removed an outlier (marked in the red circle).

Supplementary Material 2: Supplementary Fig. 2. Correlation plots depict the expression of each TE subfamily in relation to the GSVA pathway (KEGG & REACTOME) score. The pathways exhibiting the highest average correlation with the 75 TEs are illustrated, with color indicating the R value derived from Pearson correlation.

Supplementary Material 3: Supplementary Fig. 3. TE expression is correlated with IFN level in SLE in an independent dataset (PRJNA476781). (A) Correlation between the three IFN expression modules and TE score in the integrated dataset (Left: M1.2, Middle: M3.4, Right: M5.12). (B) Bubble plot shows correlation between the expression of each TE subfamily with the three IFN modules of in the integrated dataset (Left: M1.2, Middle: M3.4, Right: M5.12). (C) Heatmap showcasing the correlation between the expression of each TE subfamily and the individual genes within the three IFN modules. (D) Scatter plot shows the correlation between the subfamily level TE expression and the expression level of three IFN modules is concordant in both integrated dataset and PRJNA476781.

Supplementary Material 4: Supplementary Fig. 4. Boxplot displays a comparison of the disease activity score between patients belonging to the TE high and TE low groups (PRJNA921887). Patients in the TE high group demonstrated a significantly higher disease activity score when compared to those in the TE low group.

Supplementary Material 5.

Supplementary Material 6.

Supplementary Material 7.

## Data Availability

The data that support the findings of this study are available in Gene Expression Omnibus at https://www.ncbi.nlm.nih.gov/geo/. These data were derived from the following resources available in the public domain: PRJNA294187, PRJNA318253, PRJNA439269, PRJNA717024, PRJNA921887, PRJNA627214, PRJNA476781.

## Abbreviations

SLE: Systemic Lupus Erythematosus
IFN: Interferon
TE: Transposable Element
HERV: Human Endogenous Retrovirus
LTR: Long Terminal Repeat
LINE: Long Interspersed Element
SINE: Short Interspersed Element
ORF: Open Reading Frame
PRR: Pattern Recognition Receptor
dsRNA: Double-stranded RNA
ssRNA: Single-stranded RNA
ssDNA: Single-stranded DNA
TLR: Toll-like Receptor
GO: Gene Ontology
GSEA: Gene Set Enrichment Analysis
KEGG: Kyoto Encyclopedia of Genes and Genome
