# Supplementary figures and images for: Association of hyperactivated transposon expression with exacerbated immune activation in systemic lupus erythematosus
Source: Mob DNA. 2024 Oct 19;15:23. doi: 10.1186/s13100-024-00335-8 (PMC11490001; doi:10.1186/s13100-024-00335-8)

**A**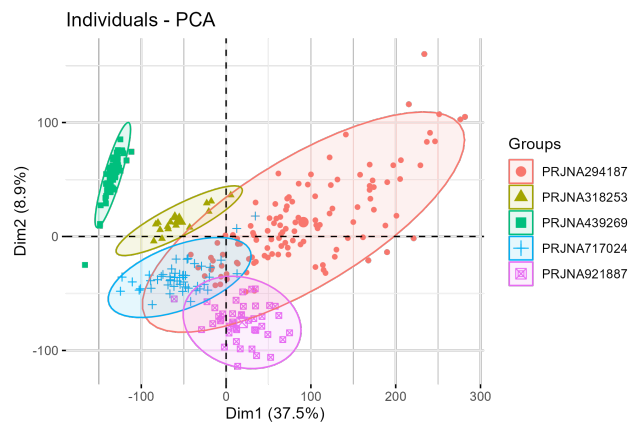**B**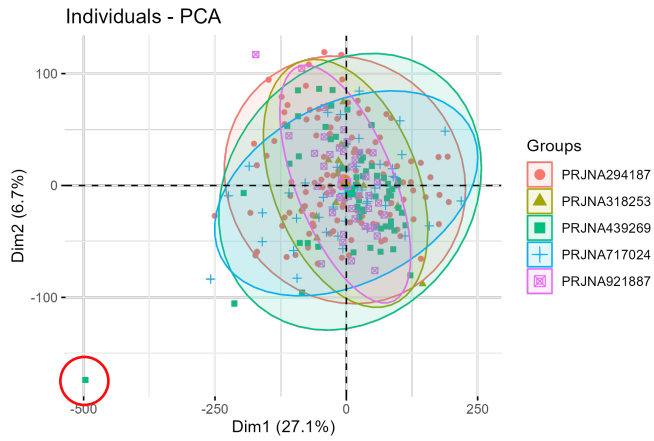**C**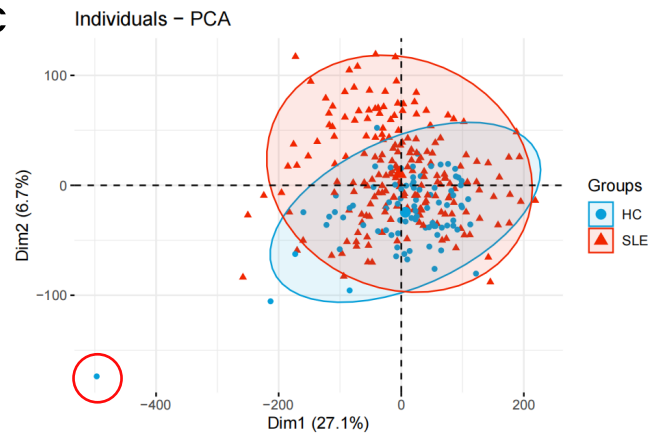**D**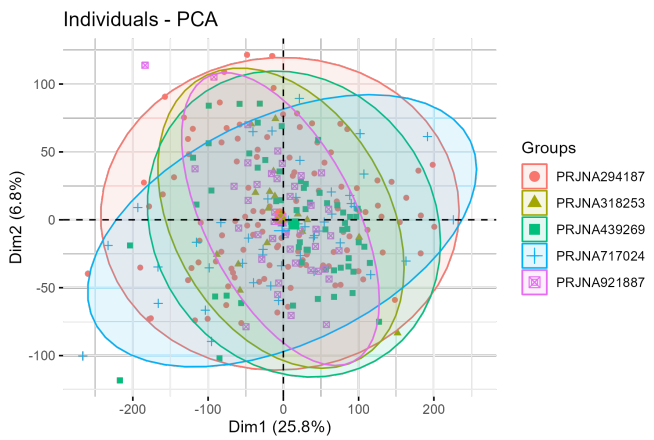**E**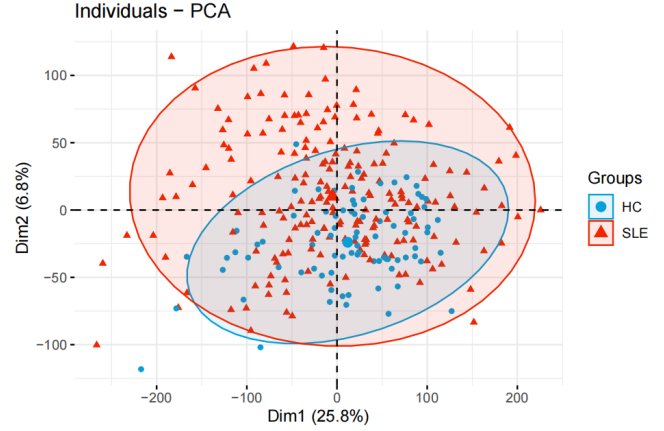

Supplement: Supplementary file 1 — Supplementary Material 1: Supplementary Fig. 1. PCA plots of all the samples from the five datasets. (A) PCA plot prior to batch correction. (B-E) Batch effect across different datasets are minimized after limma::removeBatchEffect according the PCA plots. We identified and removed an outlier (marked in the red circle). [file 13100_2024_335_MOESM1_ESM.pdf]

A

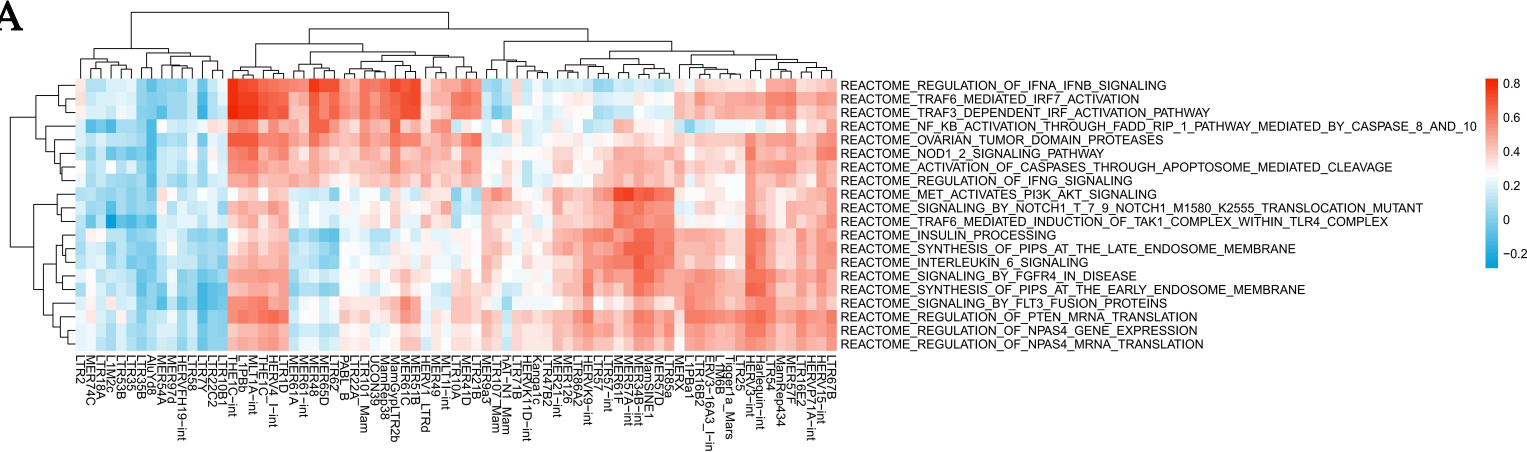

# B

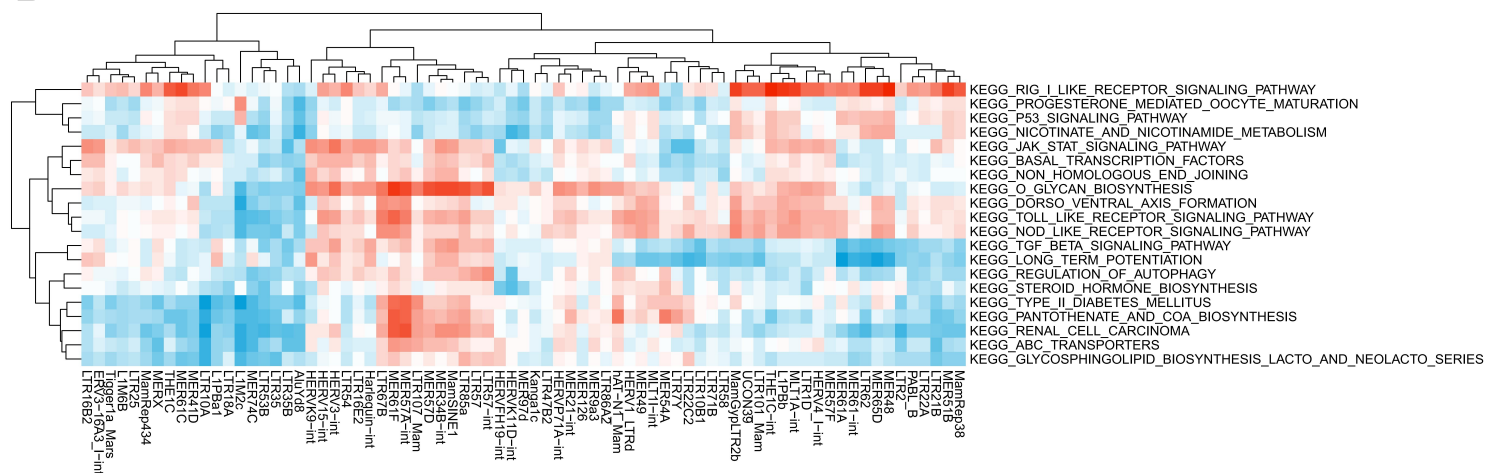

Supplement: Supplementary file 2 — Supplementary Material 2: Supplementary Fig. 2. Correlation plots depict the expression of each TE subfamily in relation to the GSVA pathway (KEGG & REACTOME) score. The pathways exhibiting the highest average correlation with the 75 TEs are illustrated, with color indicating the R value derived from Pearson correlation. [file 13100_2024_335_MOESM2_ESM.pdf]

A

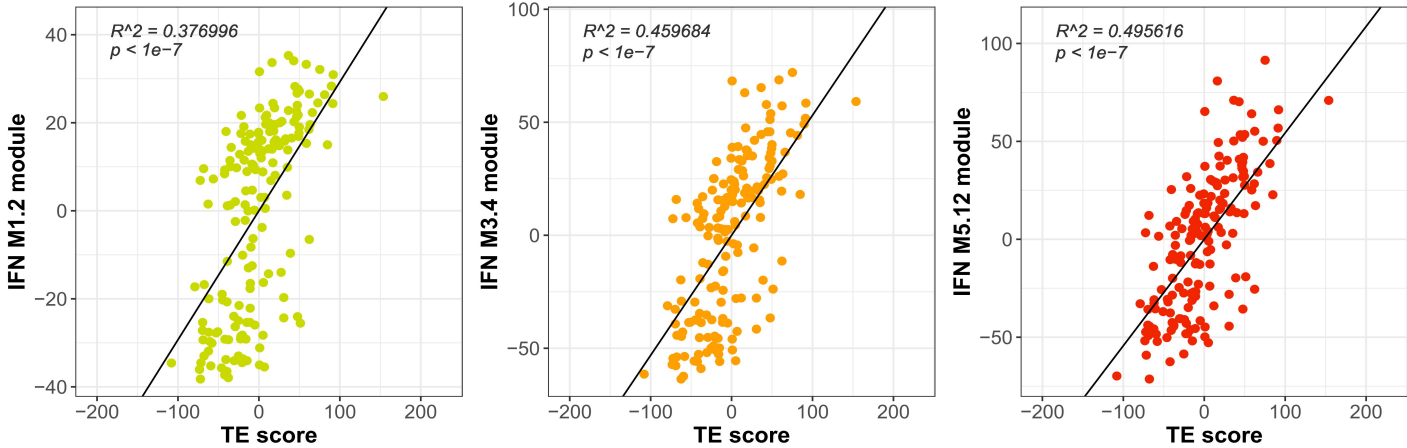

B

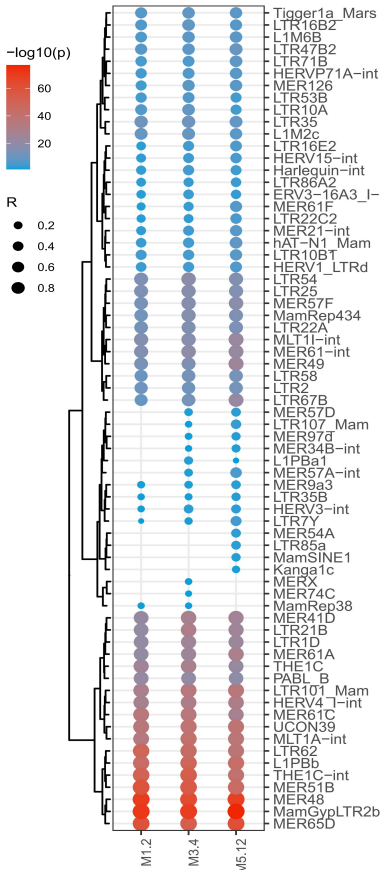

C

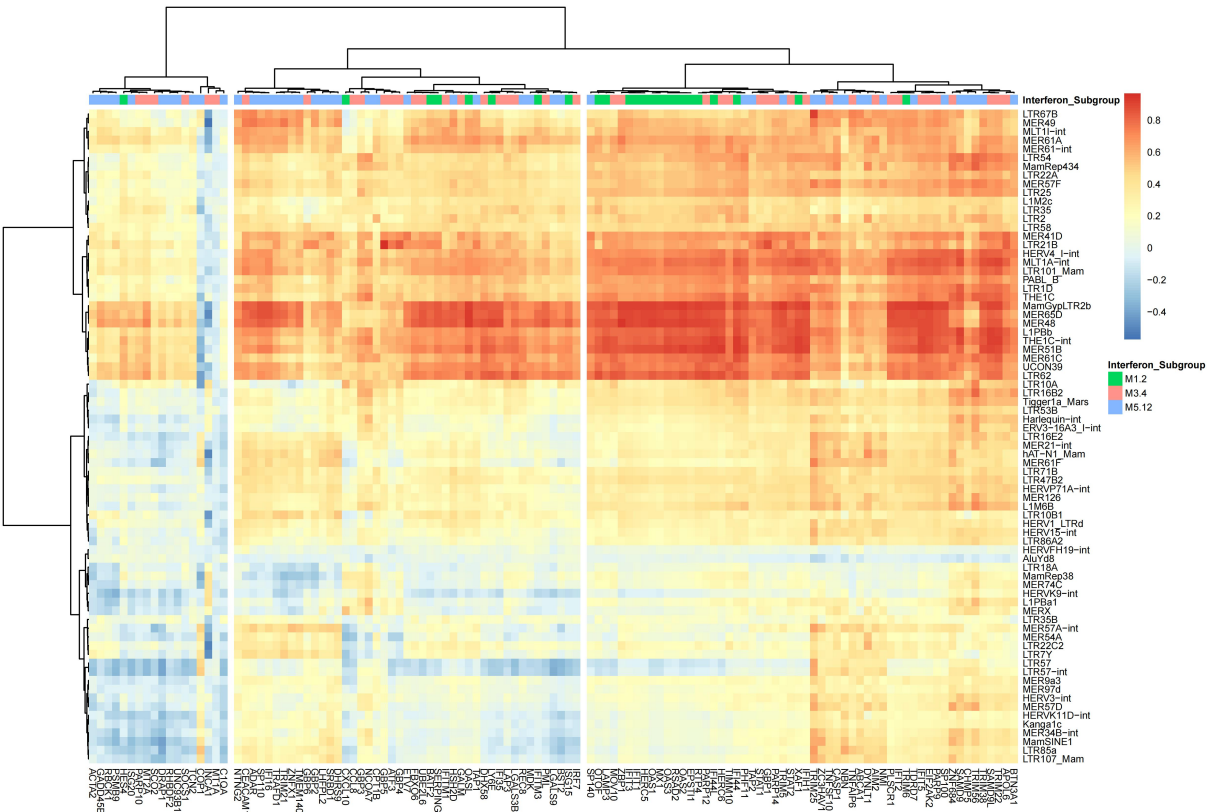

D

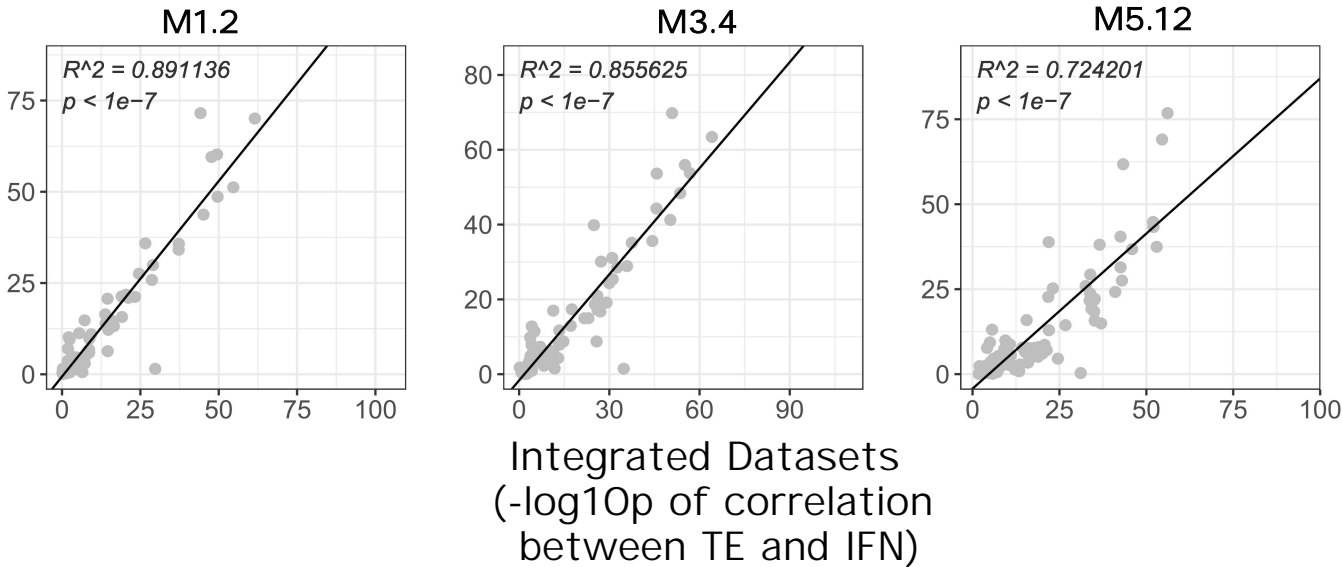

PRJNA476781  
( $-\log_{10}p$  of correlation between TE and IFN)

Supplement: Supplementary file 3 — Supplementary Material 3: Supplementary Fig. 3. TE expression is correlated with IFN level in SLE in an independent dataset (PRJNA476781). (A) Correlation between the three IFN expression modules and TE score in the integrated dataset (Left: M1.2, Middle: M3.4, Right: M5.12). (B) Bubble plot shows correlation between the expression of each TE subfamily with the three IFN modules of in the integrated dataset (Left: M1.2, Middle: M3.4, Right: M5.12). (C) Heatmap showcasing the correlation between the expression of each TE subfamily and the individual genes within the three IFN modules. (D) Scatter plot shows the correlation between the subfamily level TE expression and the expression level of three IFN modules is concordant in both integrated dataset and PRJNA476781. [file 13100_2024_335_MOESM3_ESM.pdf]

# Disease Activitiy Info From PRJNA921887

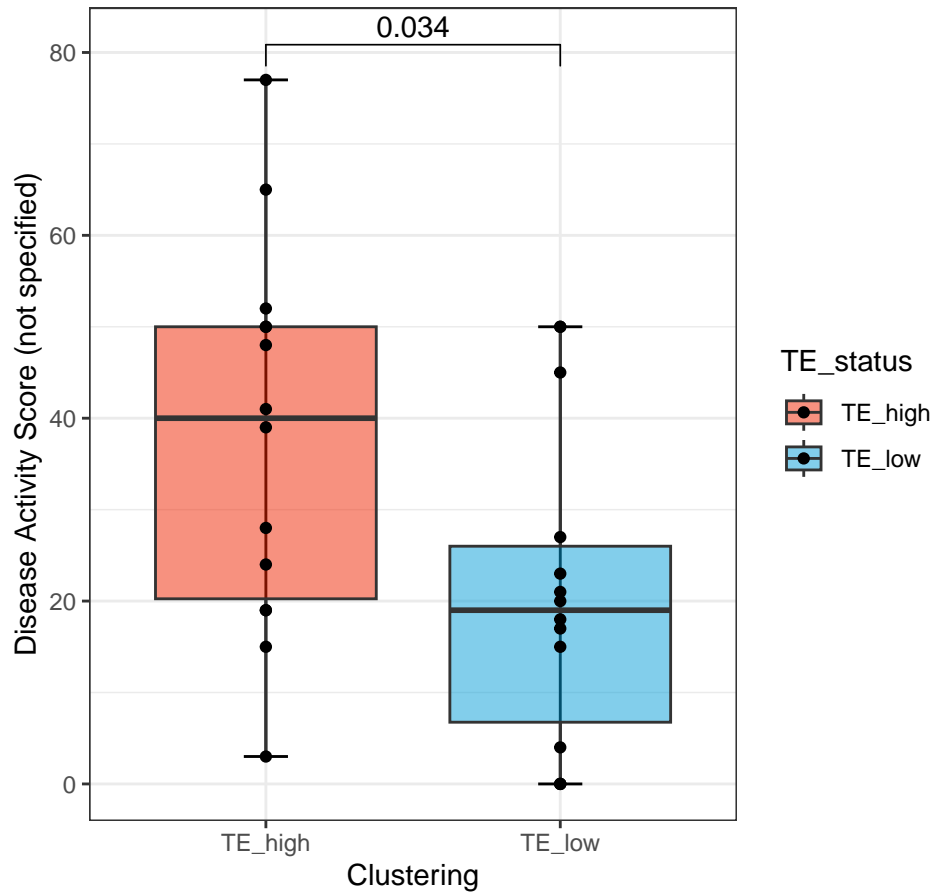

Supplement: Supplementary file 4 — Supplementary Material 4: Supplementary Fig. 4. Boxplot displays a comparison of the disease activity score between patients belonging to the TE high and TE low groups (PRJNA921887). Patients in the TE high group demonstrated a significantly higher disease activity score when compared to those in the TE low group. [file 13100_2024_335_MOESM4_ESM.pdf]
